# SERS monitoring of photoinduced-enhanced oxidative stress amplifier on Au@carbon dots for tumor catalytic therapy

**DOI:** 10.1038/s41377-022-00968-5

**Published:** 2022-09-30

**Authors:** Linjia Li, Jin Yang, Jiahui Wei, Chunhuan Jiang, Zhuo Liu, Bai Yang, Bing Zhao, Wei Song

**Affiliations:** 1 State Key Laboratory of Supramolecular Structure and Materials, College of Chemistry, Jilin University, Changchun, 130012 China; 2 Department of Vascular Surgery of China-Japan Union Hospital, Jilin University, Changchun, 130031 China; 3 College of Basic Medical Sciences, Jilin University, Changchun, 130021 China; 4 Department of Laboratory Animals, College of Animal Sciences, Jilin University, Changchun, 130062 China; 5 State Key Laboratory of Electroanalytical Chemistry, Changchun Institute of Applied Chemistry, Chinese Academy of Sciences, Changchun, 130022 China

**Keywords:** Raman spectroscopy, Plasma-based accelerators

## Abstract

Currently, artificial enzymes-based photodynamic therapy (PDT) is attractive due to its efficient capacity to change the immunosuppressive tumor microenvironment (TME). It is of great significance to study the therapeutic mechanism of novel artificial enzymes in TME through a monitoring strategy and improve the therapeutic effect. In this study, Au@carbon dots (Au@CDs) nanohybrids with a core-shell structure are synthesized, which not only exhibit tunable enzyme-mimicking activity under near-infrared (NIR) light, but also excellent surface-enhanced Raman scattering (SERS) properties. Therefore, Au@CDs show a good capability for monitoring NIR-photoinduced peroxidase-like catalytic processes via a SERS strategy in tumor. Moreover, the Au@CDs deplete glutathione with the cascade catalyzed reactions, thus elevating intratumor oxidative stress amplifying the reactive oxygen species damage based on the NIR-photoinduced enhanced peroxidase and glutathione oxidase-like activities, showing excellent and fast PDT therapeutic effect promoted by photothermal property in 3 min, finally leading to apoptosis in cancer cells. Through SERS monitoring, it is further found that after removing the NIR light source for 33 min, the reactive oxygen species (ROS) activity of the TME is counteracted and eliminated due to the presence of glutathione. This work presents a guidance to rationally design of artificial enzyme for ROS-involved therapeutic strategies and a new spectroscopic tool to evaluate the tumor catalytic therapy.

## Abstract (web summary)

Core-shell structured Au@carbon dots are fabricated to present a photoinduced-enhanced oxidative stress amplification, which can be efficiently used for tumor catalytic therapy by surface-enhanced Raman scattering technique.

## Graphical abstract

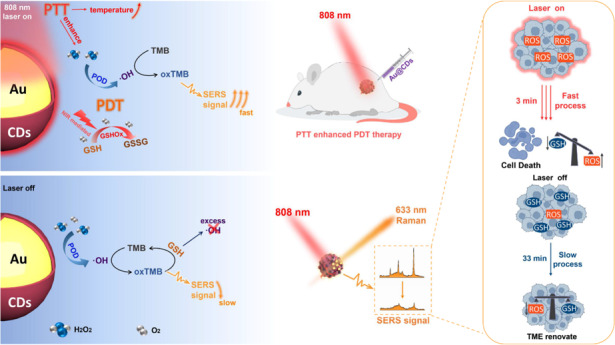

## Introduction

Mitochondrial redox homeostasis has attracted extensive research interest for cancer treatment^1,2^ because the equilibrium between reducing species^3^ and oxidizing species^4^ within tumor microenvironment (TME) plays a crucial role in majority of biological processes^1^, especially apoptotic cell death process^5^. Upon the balance of oxidizing and reducing molecules is broken by the increase of reactive oxygen species (ROS) concentration, the cancer cells will die to achieve the purpose of tumor treatment^6^. However, depleting glutathione (GSH) in TME is a challenge because the overexpressed concentration level of GSH could prevent generating toxic levels of ROS^7,8^. Thus, in the past decade, photodynamic therapy (PDT) promoted by the photothermal effect based on nanotechnology^9^ have captured comprehensive attention to target the broken of redox homeostasis in TME performed by synchronous elevating ROS levels and exhausting GSH^10,11^. However, the evidence of monitoring of oxidative stress processes based on PDT reaction promoted by photothermal effect in tumor is still lacking to insightful reveal the therapy mechanism.

Over the past few years, nanomaterials-based artificial enzymes with tunable catalytic efficiency have achieved preliminary success in antitumor immunotherapy^12^. Particularly, the noble metal-based artificial enzymes (like Au, Ag and Cu nanoparticles) allow efficient photon absorption from the ultraviolet (UV) to visible and even near-infrared (NIR) range on the basis of their characteristic plasmonic nanostructures^13^, enabling them as efficiently photocatalytic therapy materials^14^. The relevant spectral information, especially surface-enhanced Raman spectrum (SERS) will be amplified as long as the molecule is exposed to the electromagnetic field enhanced by the localized surface plasmon resonance (LSPR) effect^15,16^, providing a most sensitive route for in situ monitoring at molecular level^17^. Therefore, plasmonic nanoparticles are potential candidates for in situ monitoring of the oxidative stress processes and the therapeutic species production toward tumor eradication in real time^18–21^. However, the enzyme-mimicking activities of noble metal nanoparticles are still relatively weakened and easy to be inactivated by the aggregation effect in TME^22,23^. Therefore, the integration of noble metal nanoparticles with other nanomaterials to form new kinds of composite artificial enzymes could not only ensure the stability but also diversify the tunable enzyme-like catalytic properties^24–26^.

Herein, Au@carbon dots nanoparticles (Au@CDs NPs) with a uniform Au nucleus-CDs shell structure are fabricated via a simple chemical reduction route. Based on this unique morphological engineering, the catalytic properties and SERS capacity of Au@CDs NPs are strengthened profited from the capping agent effect of CDs and the strong charge transfer (CT) effect between Au and CDs compared with individual Au NPs. Moreover, Au@CDs express tunable peroxidase (POD) and glutathione oxidase (GSHOx)-mimicking activity based on the synergy effect between the two components in hybrid. Under NIR light irradiation, the huge amounts of hot carriers excited by surface plasma resonance (SPR) can effectively enhance enzyme-like catalytic reactions, on the other hand, the temperature rising of plasmonic structures under light excitation can further accelerate enzyme-like catalytic processes, presenting great PDT therapeutic effect promoted by photothermal property, eventually resulting in cancer cells apoptosis. Furthermore, through SERS strategies, we obtained a complete oxidative stress process in TME from 3,3’,5,5’-tetramethylbenzidine (TMB) oxidation. This work presents an insightful study about the phototherapy mechanism based on oxidative stress injury in TME, which provides the most valuable mechanism and data support for the real-time monitoring of tumor phototherapy.

## Results

### Morphology and characterization of Au@CDs

**Fig. 1:**
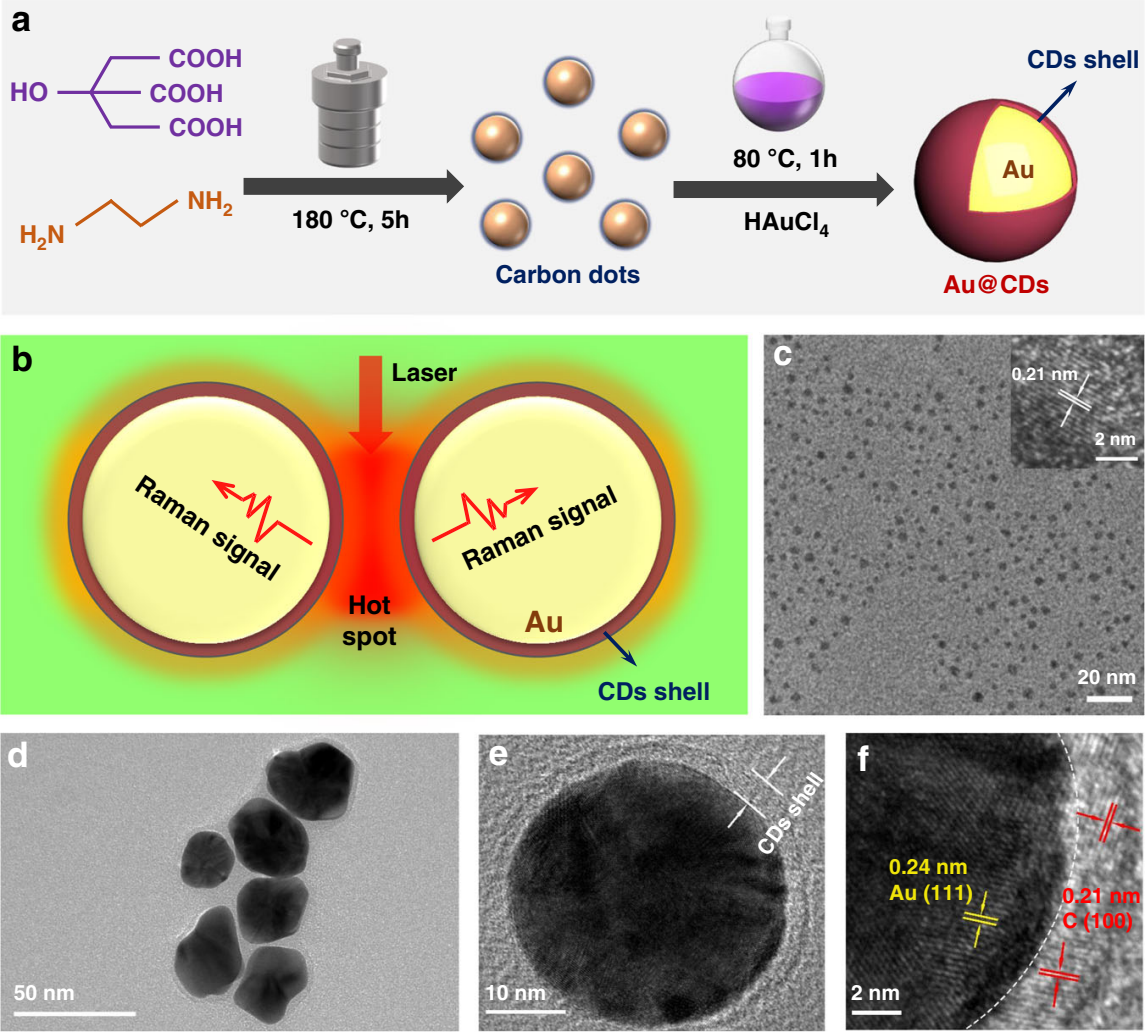
Morphology of CDs and Au@CDs. **a** The synthesis procedure of the core-shelled Au@CDs nanospheres. **b** Raman enhancement mechanism of core-shelled nanocomposites. **c** HRTEM image of initial CDs, **d**, **e** TEM image of as-prepared Au@CDs nanospheres, and **f** HRTEM image of the interface region of Au@CDs.

The synthetic protocol for core-shelled Au@CDs nanospheres is illustrated in Fig. 1a. Firstly, CDs were synthesized from ethylenediamine and citric acid via a typical hydrothermal method through sequential polymerization and carbonization processes^27^. Briefly, CDs could act as electron donor to reduce HAuCl_4_ through a chemical reduction way to generate uniform Au NPs based on the template effect^28^. In this study, the spherical Au nucleus covered with a very thin CDs layer was facilely generated in an oil bath for 1 h at 80 °C. As shown in Fig. 1b, the ultrathin CDs shell as the capping agent coating the nucleus, which prevent the aggregation of Au nanoparticles and provide a large number of stable reactive sites and Raman hot spots^21,29,30^. High-resolution transmission electron microscopy (HRTEM) was conducted to characterize the chemical structure of initial CDs and Au@CDs hybrids. It was observed that initial CDs presented a monodispersion with small sizes of only at 2–6 nm. The lattice spacing at 0.21 nm in the inset of Fig. 1c is severally closer to (100) plane of graphite carbon^31^. As shown in Fig. 1d, the prepared Au@CDs possess a unique core-shell nanostructure. The diameters of Au cores are approximate 40 nm, which are embedded tightly into an ultra-thin carbon shell with thickness of *ca*. 2 nm (Fig. 1e). The well-formed carbon shell could protect the cores from aggregation and maintain the stability. Additionally, the lattice fringes at 0.24 and 0.21 nm corresponding to (111) and (100) planes of face-centered cubic Au and graphite carbon are distinctly located at the center and edge of the spherical nanostructure (Fig. 1e)^28^, respectively, confirming the formation of core-shell structure of the sample. Furthermore, the elemental mappings of Au@CDs NPs also demonstrate that the Au cores are well wrapped by the thin carbon layer in a more visualized way (Fig. S1).

**Fig. 2:**
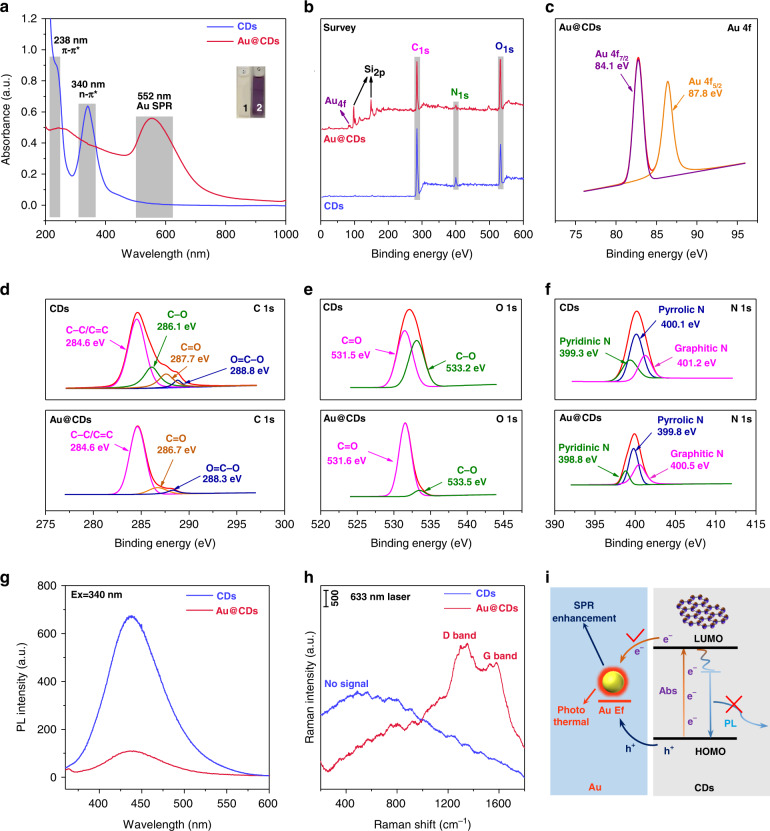
Structural characterization of CDs and Au@CDs. **a** UV-vis-NIR absorption spectra of CDs and Au@CDs in aqueous solution, (inset: the solution color of (1) CDs and (2) Au@CDs under natural light). XPS spectra of CDs and Au@CDs: **b** full survey spectrum, **c** Au 4f, **d** C 1s, **e** O 1s, **f** N 1s. **g** Photoluminescent spectra (under 340 nm excitation) and **h** Raman spectra of CDs and Au@CDs in aqueous suspension. **i** Description of the CT effect in Au@CDs hybrids.

UV-vis-NIR absorption spectra exhibit two analogous absorption peaks for both CDs and Au@CDs, one peak at 238 nm is associated with π-π* transition of sp^2^ carbon and another peak at 340 nm is assigned to n-π* transition of C=O, C-O and C-N bonds, respectively. In addition, a characteristic peak at 552 nm corresponding to SPR absorption band of Au appears in the spectrum of Au@CDs, indicating the formation of composite nanostructure. Compared with bare Au NPs (40 nm) in Fig. S2a, b, a significant red shift and extension of the SPR absorption peak was observed in Au@CDs sample, expressing a variation of the surrounding medium of Au^32^. Especially, significant shifts in the longitudinal and transverse LSPR of the Au NPs came out of the surrounding environment changing from the low altered refractive index (RI) of citric acid to the high RI of dense CDs layer^33,34^, which can promote the optical absorption capacity in the long-wavelength light region. As shown in Fig. S2c–f, the dense CDs shell with negative charge avoids the accumulation of Au nucleus and thus ensures the uniform dispersion and stability of Au@CDs NPs hybrid system for a long time. Moreover, the chemical composition of CDs and Au@CDs were also characterized by X-ray photoelectron spectroscopy (XPS) measurement (Fig. 2b–f). The full survey spectrum of Au@CDs shows typical peaks of C, N, and O elements of CDs layer and additional Au peaks, while the Si signals root in the silicon substrate. For the individual CDs, the detailed information about the elemental states were presented in Fig. 2d–f. The C 1s spectrum can be deconvoluted into four main sub peaks at 284.6, 286.1, 287.7 and 288.8 eV, which are assigned to C=C/C-C, C-O, C=O and O=C-O groups (Fig. 2d). For O 1 s spectrum in Fig. 2e, the main peak is composed of two parts of composition at 531.5 and 533.2 eV, corresponding to C=O and C-O groups. In Fig. 2f, three types of pyridinic N, pyrrolic N, and graphitic N were observed at 399.3, 400.1, and 401.2 eV, respectively^31,35^. All the above results corresponded with the FTIR information of CDs in Fig. S3. Specifically, the peaks at 3785–3416 cm^−1^ are observed, implying a majority of O-H/N-H containing groups on the surface of CDs. Furthermore, the stretching vibrations of C-H at 1384, 2893 and 2950 cm^−1^, bending vibrations of N-H at 1555 cm^−1^, and the vibrational absorption band of C=O at 1665 cm^−1^ are also observed. Besides, the peak at 1116 cm^−1^ corresponding to C-NH-C stretching vibration demonstrates the presence of different chemical states of C, N and O atoms on CDs surface^27^. In the Au@CDs hybrid system, a pair of peaks at 84.1 (Au 4f_7/2_) and 87.8 eV (Au 4f_5/2_) associated with the binding energies of the Au^0^ appear in Fig. 2c, confirming the formation of Au nucleus in the hybrid^24^. Significantly, as shown in Fig. 2d, e, the signals of the ratio of C-OH dropped dramatically in the XPS spectrum of Au@CDs compared with that of CDs, further testifying the reductive ability of functional groups (especially -OH) of CDs for reducing Au^3+^ to form Au@CDs NPs. Detailed information of different group types of CDs and Au@CDs has been expressed in Tables S1 and S2, which may lead to a deeper understanding of the formation mechanism of Au@CDs. Moreover, the existence of another oxygen containing functional groups (e.g., C=O and O=C-O) of Au@CDs might be beneficial to catalytically decompose H_2_O_2_ to generate ·OH^36,37^.

In this work, the as-prepared CDs exhibit typical excitation-dependent photoluminescence (PL) behavior in Fig. S4 because of the unique surface state^38^. However, because of the CT effect, the excited electrons from the lowest unoccupied molecular orbital level (LUMO) of CDs will transfer to the Fermi level (E_f_) of Au nucleus in the Au@CDs system, hindering the returning of electrons to the ground state, resulting in a fluorescence quenching in Au@CDs hybrid (Fig. 2g). Meanwhile, the generated holes from highest unoccupied molecular orbital level (HOMO) of CDs will also transfer to the E_f_ of Au and achieve a recombination there, which could produce more intense SPR and enhance the photothermal effect of Au@CDs. Owing to the strong fluorescence of bare CDs, it is difficult to reveal their chemical structures by Raman spectroscopy (Fig. 2h). However, benefitting from strong quenching fluorescence effect, the characteristic signals of D band (1350 cm^−1^) and G band (1580 cm^−1^) of carbon materials corresponding to the disordered and aromatic domains can be clearly observed in the Raman spectrum of Au@CDs sample. Furthermore, the ratio of I_D_/I_G_ for Au@CDs is around 1.17, revealing the defect characteristic of this carbon materials with the ability of donating or withdrawing electrons^21^.

### Photothermal effect evaluation of Au@CDs

**Fig. 3:**
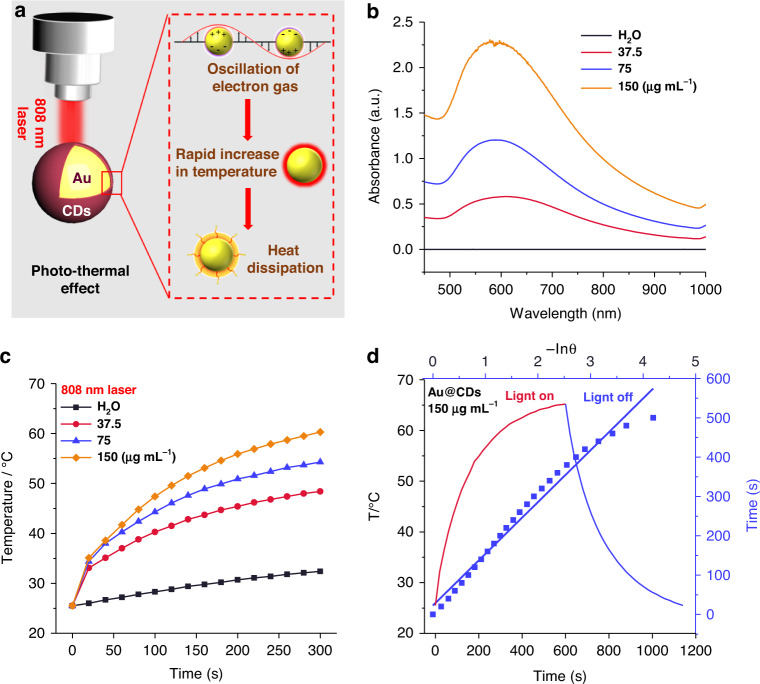
Photothermal effect evaluation of Au@CDs. **a** Mechanism of the photo-thermal process induced by the SPR effect of Au@CDs, **b** vis-NIR absorbance spectra of Au@CDs in aqueous solution with varied concentrations, **c** the heating curves of Au@CDs under irradiation of 808 nm laser at varying concentrations in water (laser power: 2 W cm−2), **d** the heating and cooling curves of Au@CDs for laser on/off and plot of cooling time versus the negative natural logarithm of the temperature driving force.

As shown in Fig. 3a, when the SPR effect takes place, quite a few of the excited hot electrons are cooled in a short time due to the electron-phonon interaction, then the metal lattice can be heated, resulting in a photothermal conversion of the plasmon^39–42^. As aforementioned, the SPR absorption peak of Au@CDs was broadened and moved to the NIR region to present strong photothermal effect upon core-shelled nanostructure generation. The absorbance spectra of Au@CDs with different concentrations presented a desired absorption at 800–1000 nm (Fig. 3b). Due to the deeper light penetration, photothermal therapy with absorbance in the NIR-I window (700–900 nm) is very attractive^43^, which could be related to the novel core-shelled nanostructure and the intense charge transferring interaction between Au and CDs. In addition, the absorption intensity of Au@CDs increases along with the concentration through the Lambert-Beer law. Next, the photothermal effects of Au@CDs were explored. As shown in Fig. 3c, the rate of temperature rises under the NIR laser irradiation and the final temperature correlated with the Au@CDs concentration. Since pure water presented small temperature rising under the same condition, this result illustrates the eminent capacity of Au@CDs to convert NIR light energy into heat. Subsequently, another vital factor for exploring the potential of Au@CDs as photothermal activity in NIR region is the photothermal conversion efficiency. As the heating and cooling curves of Au@CDs expressed in Fig. 3d, the photothermal conversion efficiency of Au@CDs was estimated to be 39.96% according to previously reported method^44,45^, which is much higher than he individual Au NPs with 40 nm (17%) in Fig. S5, demonstrating that the photothermal effect can be improved tremendously based on the carbon shell. In addition, the comparative analysis of photothermal activities based on different types of plasmon-based nanomaterials with the prepared Au@CDs has been shown in Table S3, further expressing the superiority of Au@CDs for cancer photothermal therapy.

**Fig. 4:**
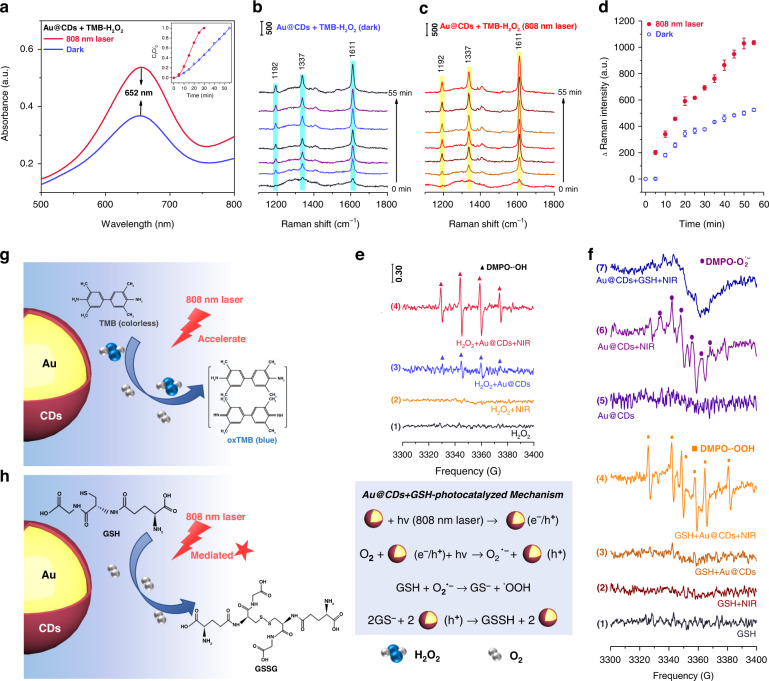
Photoinduced enhanced enzyme-like catalytic properties of Au@CDs. **a** UV–vis spectra of Au@CDs+ H_2_O_2_ +TMB systems in darkness or under 808 nm laser irradiation (react for 30 min), **b** SERS monitoring of Au@CDs+ H_2_O_2_ +TMB systems in darkness and **c** under 808 nm laser irradiation. **d** SERS intensities of peaks at 1611 cm^–1^ versus times. **e** EPR spectra of varied systems: (1) H_2_O_2_, (2) H_2_O_2_ + NIR, (3) H_2_O_2_ + Au@CDs, (4) H_2_O_2_ + Au@CDs + NIR. DMPO was used as spin adduct and NIR light is from 808 nm laser. **f** EPR spectra of varied systems: (1) GSH, (2) GSH + NIR, (3) GSH + Au@CDs, (4) GSH + Au@CDs + NIR in aqueous solution and (5) Au@CDs, (6) Au@CDs + NIR, (7) GSH + Au@CDs + NIR in methanol solution. DMPO was used as spin adduct and NIR light is from 808 nm laser. **g** Possible mechanism for the photoinduced enhanced POD-mimicking activities and **h** the photo-mediated GSHOx-mimicking activities under 808 nm laser irradiation.

### Photoinduced enhanced enzyme-like catalytic properties of Au@CDs

CDs-based nanocomposites usually display desirable catalytic properties because of the unique surface state energy levels and a large number of functional groups^46^. Moreover, both of the photoexcited hot carriers and photothermal effects from SPR effect can mediate or enhance the catalytic activity under light irradiations in the plasmon mediated catalytic reactions (PMCRs)^47^. In the core-shelled nanostructured hybrid system of Au@CDs, the presence of multiple functional groups on the CDs shell provides plenty of active sites for donating and withdrawing electrons^36,37^, meanwhile, the Au cores with typical plasmon effect can further promote the catalytic processes under optical excitation, thus the synergistic effect may improve the catalytic activity in the hybrid system.

In our previous works, noble metal/CDs-composite nanomaterials have presented to show a certain peroxidase (POD)-like activity^21,30^. In Fig. 4a, the typical UV-vis absorption spectrum shows that an excelelnt POD-like activity was achieved for our prepared Au@CDs, which can oxidize 3,3’,5,5’-tetramethylbenzidine (TMB) to present an absorption peak at 652 nm. In comparison, significant changes of absorption indentisy was obtained in the same system under 808 nm laser irradiation in 30 min. Real-time UV-vis spectra expressed that the intensity at 652 nm increases and almost reaches maximum over time in the darkness of Au@CDs + H_2_O_2_ + TMB system, indicating that most of the TMB molecules have been transformed to oxidized TMB (oxTMB) in 55 min (Fig. S6a and inset of Fig. 4a). By contrast, the time to complete the above catalytic process is reduced to 30 mins by NIR light irradiation in Fig. S6b, displaying a much higher POD-like activity. These results demonstrate that the Au@CDs exhibit unique photoinduced enhanced POD-like activity.

The Au@CDs also present a remarkable SERS property based on the enhanced plasmonic activity and abundant hot spots involoving the core-shelled morphological engineering (Fig. 1b)^29^. In Fig. S7a, the SERS spectra of crystal violet (CV) moleucules were proived to show that CV with a low concentration of 10^–8 ^M can still be detected, demonstrating the outstanding SERS property. The details of spectral bands of CV molecular are consistent with previous studies in Table S4. By contrast, the SERS activity of the Au@CDs substrate is much better than that of individual Au NPs (Fig. S7c), which might resulting from the improved absorption of CV with CDs via the electrostatic interactions and the enhanced electromagnetic field from Au nuclues/CDs interactions. Thus, the Au@CDs + H_2_O_2_ + TMB system can be used to monitor the POD-like reaction via the SERS strategy. Figure 4b, c show the SERS monitoring processes of this system in dark and under 808 nm laser irradiation, both of them showed characteristic signals of oxTMB at 1192, 1337, 1611 cm^−1^, which are consistent with previous study^30^. Similar to the results obtained from UV-vis spectroscopy means, NIR laser irradiation can greatly accelerate the signal intensity increasing rate of the oxidation products (Fig. 4d). Meanwhile, the mechanism of POD-like activity of Au@CDs is investigated by electron paramagnetic resonance (EPR) measurement in Fig. 4e. Specifically, the appearing signals with a certain relative peak magnitude corresponding to 5,5-Dimethyl-1-pyrroline N-oxide (DMPO)-·OH demonstrate the generation of ·OH in Au@CDs + H_2_O_2_ system^48^, which might be resulted from the decomposion of H_2_O_2_ through the following Fenton-like reactions (1) – (3):

1 $${{{\mathrm{Au}}}}@{{{\mathrm{CDs}}}} + {{{\mathrm{H}}}}_2{{{\mathrm{O}}}}_2 \to {{{\mathrm{Au}}}}@{{{\mathrm{CDs}}}}\left( {{{{\mathrm{e}}}}^ - } \right){{{\mathrm{ + }}}} \cdot {{{\mathrm{OOH}}}} + {{{\mathrm{H}}}}^ +$$

2 $${{{\mathrm{Au}}}}@{{{\mathrm{CDs}}}} + \cdot {{{\mathrm{OOH}}}} \to {{{\mathrm{Au}}}}@{{{\mathrm{CDs}}}}\left( {{{{\mathrm{e}}}}^ - } \right) + {{{\mathrm{H}}}}^ + + {{{\mathrm{O}}}}_2$$

3 $${{{\mathrm{Au}}}}@{{{\mathrm{CDs}}}}\left( {{{{\mathrm{e}}}}^ - } \right) + {{{\mathrm{H}}}}_2{{{\mathrm{O}}}}_2 \to {{{\mathrm{Au}}}}@{{{\mathrm{CDs}}}} + {{{\mathrm{OH}}}}^ - + \cdot {{{\mathrm{OH}}}}$$

Under the NIR light, Au@CDs can produce more plasmonic-carriers to participate in the reaction. On the other hand, the photothermal effect of Au@CDs can further accelerate the carriers transport, providing a higher peak intensity under NIR light irradiation than in darkness, which can be resulting from the photoinduced enhanced effect (Fig. 4g).

We next explored the photo-mediated glutathione oxidase (GSHOx)-mimicking activity of Au@CDs, which can produce oxidized glutathione (GSSG) from reduced glutathione (GSH). As shown in Fig. 4f, no obvious signal is observed from individual GSH with or without NIR light irradiation. Additionally, the reaction between GSH and Au@CDs is so faint in darkness so that no significant changes in EPR spectrum have been observed. It is noteworthy that there are six groups of peaks which are not completely symmetrical appear in the Au@CDs + GSH + NIR system, which can be ascribed to trapped ·OOH radicals^49^. In order to reveal the source of the above signals, we observed that Au@CDs can generate O_2_^·−^ radicals under NIR irradiation in methanol solution, since it is difficult to obtain the signal of O_2_^·−^ radicals in aqueous solution, and the signal of O_2_^·−^ radicals were completely quenched upon GSH was added to the system. Based on the previous reports, we speculate that the reaction mechanism is as follows:

4 $${{{\mathrm{Au}}}}@{{{\mathrm{CDs}}}} + {{{\mathrm{hv}}}}\left( {{{{\mathrm{808}}}}\,{{{\mathrm{nm}}}}\,{{{\mathrm{laser}}}}} \right) \to {{{\mathrm{Au}}}}@{{{\mathrm{CDs}}}}\left( {{{{\mathrm{e}}}}^ - /{{{\mathrm{h}}}}^ + } \right)$$

5 $${{{\mathrm{O}}}}_2 + {{{\mathrm{Au}}}}@{{{\mathrm{CDs}}}}\left( {{{{\mathrm{e}}}}^ - /{{{\mathrm{h}}}}^ + } \right) + {{{\mathrm{hv}}}} \to {{{\mathrm{O}}}}_2^{\cdot - } + {{{\mathrm{Au}}}}@{{{\mathrm{CDs}}}}\left( {{{{\mathrm{h}}}}^ + } \right)$$

6 $${{{\mathrm{GSH}}}} + {{{\mathrm{O}}}}_2^{ \cdot - } \to {{{\mathrm{GS}}}}^ - + \cdot {{{\mathrm{OOH}}}}$$

7 $$2{{{\mathrm{GS}}}}^ - + 2{{{\mathrm{Au}}}}@{{{\mathrm{CDs}}}}\left( {{{{\mathrm{h}}}}^ + } \right) \to {{{\mathrm{GSSH}}}} + 2{{{\mathrm{Au}}}}@{{{\mathrm{CDs}}}}$$

All these results give evidence of the POD- and GSHOx-like activities of Au@CDs associated with GSH depletion and ROS generation, respectively (Fig. 4g, h). Interestingly, the catalytic performances of the two reactions have been greatly enlarged under NIR light irradiation due to the plasmonic effect of Au@CDs, and the above abilities would allow continuous generation of ROS in the TME.

### Therapeutic effect of Au@CDs and SERS monitoring of the catalytic reaction in the TME

Firstly, we evaluate the cytotoxicity of varied concentrations of Au@CDs towards tumor cells (4T1), cell viability was quantified through cell counting kit-8 (CCK-8). Obviously, the cell viability decreases with the increasing of the Au@CDs concentration (Fig. S8a). The cell viability of the Au@CDs solution with a concentration of 160 μg mL^−1^ achieves around 30% after 24 h of incubation under dark conditions. Subsequently, a lower concentration (20 μg mL^−1^) was selected to investigate the in vitro therapeutic capability of Au@CDs against tumor cells based on photoinduced enhanced enzyme-like catalytic properties. Fig. S8b shows in vitro antitumor activities to 4T1 cells after different treatments. By contrast, it was found that the cell viability did not decrease significantly in the groups treated with only Au@CDs or 808 nm laser irradiation. However, for the groups treated with H_2_O_2_, ROS generation is significantly enhanced and can be further boosted treated with both H_2_O_2_ and Au@CDs, and cell viability is reduced to 31%, which is explained by the POD-like activity of Au@CDs. As a result of the photoinduced enhanced enzyme-like catalytic properties of Au@CDs, the cell viability of Au@CDs + H_2_O_2_ system under 808 nm laser treatment is markedly reduced to 19%. The further improvement of antitumor activity results from the consumption of intracellular GSH and the photothermal enhancement of Au@CDs. The elevation of intracellular ROS levels can cause cell death through apoptosis and direct necrosis pathways. In this study, 4T1 cell apoptosis and necrosis ratios of blank and the Au@CDs + H_2_O_2_ + 808 nm laser treatment groups within the 24 h incubation period were determined. As shown in Fig. S9, the Au@CDs + H_2_O_2_ + 808 nm laser treatment only leads to direct necrosis of 4T1 cells and has little relation to cell apoptosis.

**Fig. 5:**
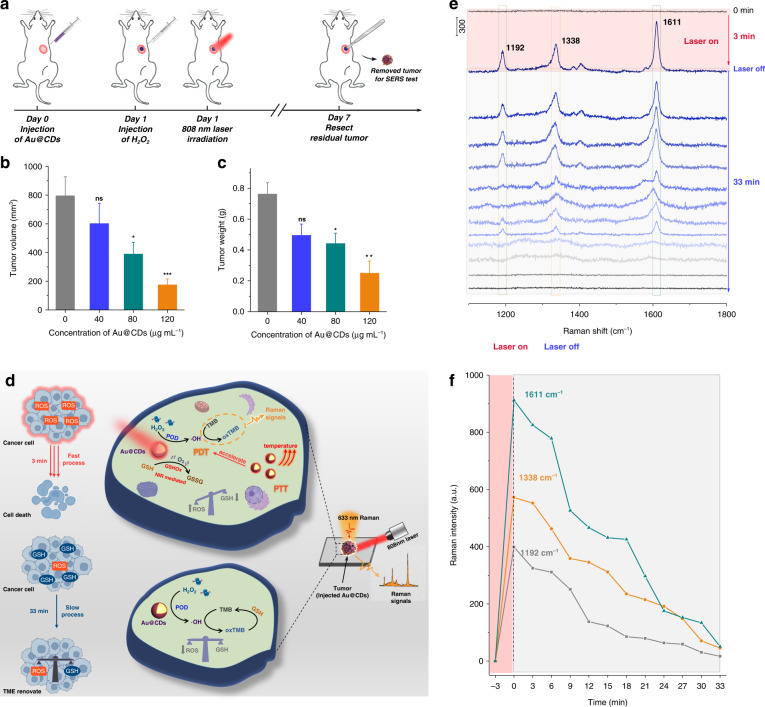
Therapeutic effect of Au@CDs and SERS monitoring of the catalytic reaction in the TME. **a** Treatment schedule for in vivo anti-metastasis study. **b** Tumor volume and **c** tumor weight in mice after Au@CDs + H_2_O_2_ + 808 nm laser treatments (2 W cm^−2^) with different concentration of Au@CDs. **d** Illustration of Raman studies of intratumor oxidative stress processes. **e** Time-dependent SERS spectra during the process of oxidative stress in tumor and **f** time-dependent intensities of the characteristic bands of oxTMB.

Following the treatment schedule in Fig. 5a, further in vivo cancer treatment was conducted with breast cancer tumor bearing mice. The mice, which have been injected of H_2_O_2_ and Au@CDs with different concentrations were irradiated with NIR laser (10 min) at 24 h post injection. In Fig. 5b, c, comparing with the control group, tumor growth was significantly inhibited with the increasing concentration of Au@CDs within 7 days, resulting from the integration of PDT and photothermal effect. The tumor photos also express the therapeutic effect of Au@CDs + H_2_O_2_ + 808 nm laser treatment in Fig. S10. Furthermore, achieving and characterizing of intratumor oxidative stress behavior and the study of catalytic processes about the reactive oxygen species damage to the tumor are of vital importance for studying the therapy mechanisms of artificial enzymes. Due to the unique SERS ability of Au@CDs, intrinsic Raman signals of Au@CDs NPs have been obtained on the resected tumors after PDT therapy processes. In Fig. S11, the same Raman signals about the D and G bands of Au@CDs can be observed as the results in Fig. 2h, proving that the structure of Au@CDs still remained intact after in vivo PDT therapy. When H_2_O_2_ and TMB were added to the removed tumor after therapy, no obvious Raman signal was generated without 808 nm laser irradiation. Remarkably, Raman signals attributed to oxTMB emerged rapidly once the 808 nm laser irradiated the sites of Au@CDs aggregation, and the spectral bands at 1192, 1337 and 1611 cm^−1^ related to the oxTMB were clearly visible in Fig. 5e. It is indicated that photoinduced enhanced enzyme-like catalytic reactions mediated by 808 nm laser plays a main factor in increasing ROS level in TME. Specifically, the enhanced effect of photothermal property under NIR laser irradiation for PDT processes can catalyze the decomposition of H_2_O_2_ and the decrease of GSH level in a very short time, further amplifying the reactive oxygen species damage to lead tumor elimination. Subsequently, the signal of oxTMB decrease over 33 min upon turning off the NIR laser, which is very slow compared to the increase process mentioned above under laser irradiation (3 min) (Fig. 5e, f). This result further suggests that overexpressed reductants (such as GSH) in TME will consume over-expressed ROS again, then until the ROS activity is counteracted and eliminated, the oxidation-reduction equilibrium state will be realized again.

## Discussion

In conclusion, unique Au@CDs hybrid were successfully prepared to exhibit dual characters of strong SERS enhancement and excellent photoinduced enhanced enzyme-like catalytic properties. Owing to the formation of CDs shell, the prepared Au@CDs NPs display a redshifted SPR absorption and excellent photothermal activity under 808 nm laser irradiation induced by the plasmon. Compared with conventional nanomedicines and immunotherapeutic agents, Au@CDs possess the advantages of the PDT therapeutic effect promoted by photothermal property in cascade reactions to elevate intratumor oxidative stress by GSH depletion and ROS generation, eradicating tumors by immunogenic cell death. Furthermore, through SERS strategies, we obtained a complete oxidative stress process in TMB, expressed an insightful study about the reaction mechanisms, which provides the most valuable mechanism and data support for the real-time monitoring of tumor phototherapy and normal tissue self-repairing.

## Materials and methods

### Synthesis of carbon dots-supported Au nanoparticles (Au@CDs NPs)

A mixed 30 mL of aqueous solution consisting of HAuCl_4_ (0.4 mmol L^−1^) and CDs (33 mg L^−1^) was kept at a temperature of 80 °C in an oil bath for 1 h until a stable dark fuchsia suspension was produced. Then, the suspension was centrifuged and washed with deionized water to remove the excess CDs. The optimized nanoparticles are core-shell structures with a mean diameter of ca. 40 nm. And the sample was redispersed in 10 mL deionized water.

### In vivo therapeutic effect of Au@CDs and SERS monitoring of the catalytic reaction in the TME

The study protocol (No. SY20212006) was approved by the laboratory animal center of Jilin University and was performed following the guidelines and regulations established by the tab of animal experimental ethical inspection, JLU.

Mice were received subcutaneous injection of mouse mammary tumor cells (1 × 10^7^ cells). When the sizes of the tumors reached ~50 mm^3^, they were divided into four groups (*n* = 5 in each group): (1) saline, (2) Au@CDs (40 μg mL^−1^ at the dosage of 0.1 mg kg^−1^) + H_2_O_2_ + NIR light, (3) Au@CDs (80 μg mL^−1^ at the dosage of 0.2 mg kg^−1^) + H_2_O_2_ + NIR light, (4) Au@CDs (120 μg mL^−1^ at the dosage of 0.3 mg kg^−1^) + H_2_O_2_ + NIR light. At 23 h post-injection, mice in group (2), (3), (4) were injection 50 μL H_2_O_2_ (10^–3 ^M) and at 24 h post-injection, above groups were exposed to 808 nm NIR light irradiation (2 W cm^–2^) for 5 min. Residual tumors were surgically resected on day 7.

Above residual tumors (cut from the mice) in group (3) were collected for the SERS tests. The tumors were injected with a mixture solution (50 μL) of H_2_O_2_ (5 mM) and TMB (15 mM) and irradiated with an 808 nm laser. Then, the tumors were directly placed under the LabRAM ARAMIS Smart Raman Spectrometer to observe the SERS signals over time and the excitation source was the 633 nm line of a He/Ne laser.

## Supplementary information

Supplemental material
