# Supplementary material for: SERS monitoring of photoinduced-enhanced oxidative stress amplifier on Au@carbon dots for tumor catalytic therapy
Source: Light Sci Appl. 2022 Sep 30;11:286. doi: 10.1038/s41377-022-00968-5 (PMC9525678; doi:10.1038/s41377-022-00968-5)
Supplement: Supplementary file 1 — Supplemental material [file 41377_2022_968_MOESM1_ESM.docx]

#### Supporting Information

**SERS monitoring of photoinduced-enhanced oxidative stress amplifier on Au@carbon dots for tumor catalytic therapy**

Linjia Li^1^, Jin Yang^3^, Jiahui Wei^4^, Chunhuan Jiang^5^, Zhuo Liu^2*^, Bai Yang^1^, Bing Zhao^1*^, Wei Song^1*^

^1^State Key Laboratory of Supramolecular Structure and Materials, College of Chemistry, Jilin University, Changchun, 130012, China;

^2^Department of Vascular Surgery of China-Japan Union Hospital, Jilin University, Changchun 130031, China;

^3^College of Basic Medical Sciences, Jilin University, Changchun 130021, China;

^4^Department of Laboratory Animals, College of Animal Sciences, Jilin University, Changchun 130062, China;

^5^State Key Laboratory of Electroanalytical Chemistry, Changchun Institute of Applied Chemistry, Chinese Academy of Sciences, Changchun 130022, China;

*E-mail: lzhuo@jlu.edu.cn (Z. L), zhaob@jlu.edu.cn (B. Z), weisong@jlu.edu.cn (W. S.).

### Materials

Chloroauric acid (HAuCl_4_·4H_2_O) and ethylenediamine were bought from Sinopharm Chemical Reagent Co., Ltd. Citric acid(C_6_H_8_O_7_·H_2_O) and H_2_O_2_ (30%) were obtained from Beijing Chemical Works, Crystal violet (CV) and 3,3’,5,5’-tetramethylbenzidine (TMB) were bought from Sigma-Aldrich corporation. Glutathione (GSH) and 5,5-Dimethyl-1-pyrroline N-oxide (DMPO) were obtained from Aladdin.

### Characterization

The morphologies of CDs, Au@CDs and Au NPs were observed on a JEOL JEM-2100F transmission electron microscope (TEM) operated at 200 kV. UV-vis spectra were obtained from Shimadzu UV-3600 UV-Vis-NIR spectrophotometer. The fluorescence spectra were recorded on Shimadzu 5301PC fluorescence spectrometer. X-Ray photoelectron spectroscopy (XPS) data were obtained from a Thermo ESCALAB 250 photoelectron spectrometer with Al Ka X-ray radiation. Electron paramagnetic resonance (EPR) measurement was carried out on BRUKER E500 EPR spectrometer at room temperature. The Raman spectra for CV moleculesand Raman spectra for monitoring the catalytic reactions were measured with by a LabRAM ARAMIS Smart Raman Spectrometer with the radiation from an air-cooled He/Ne laser (633 nm), the laser power at the sample position was typically 3.6 mW.The spectrometer was calibrated by Raman band at 520.7 cm^-1^ of a Si wafer.In the photocatalytic experiments, the 808 nm NIR laser device (LE-LS-808-5000TFCA) purchased from Lieu Optoelectronics Co., LTD was used as the NIR light source.

### Synthesis of carbondots

CDs were prepared with the similar procedure based on the previous report^1^. In detail, citric acid (1.0507 g) and ethylenediamine (335 µL) was dissolved in 10 mL deionized water. Then the solution was transferred to a poly (tetrafluoroethylene) (Teflon)-lined autoclave and heated at 180 ºC for 5 h. After the reaction, the reactors were cooled to room temperature. The brown-black and transparent product wassubjected to dialysis in order to obtained the CA-EDA carbondots (CDs) powders.

### Preparation of individual Au nanoparticles by sodium citrate.

Au NPs with an average diameter of ca. 40 nm was prepared by the reduction of an aqueous solution of HAuCl_4_ with sodium citrate, which has been reported by G. Frens^2^: Typically, 2.42 mL of 1 wt%HAuCl_4_ solution was added into 200 mL water. After the solution wasboiled, 3 mL of 1 wt% sodium citrate solution was injected into the solution to obtain Au NPs with a diameter of 40 nm. Finally, a certain amount of the Au NPs were redispersed in deionized water to prepare Au NPs suspension.

**SERS properties of Au@CDs and individual Au NPs**

The CV was used as a Raman probe to estimate the SERS properties of Au@CDs and Au NPs. In a typical experiment, the Au@CDs or Au NPs aqueous solution (0.45 mL) were dispersed in 50 μL of CV solution (final concentration:10^-4^-10^-8^ M) over the course of 45 min for the SERS measurements. The SERS experiments were performed with a LabRAM ARAMIS Smart Raman Spectrometer and the excitation source was the 633 nm line of a He/Ne laser.

### Photothermal transformation properties of Au@CDs under 808 nm laser irradiation

Firstly, 1 mL Au@CDs aqueous solutions with different mass concentrations were placed in a quartz colorimetric dish with a base area of 1 cm×1 cm, and 808 nm laser with a power of 2 Wcm^-2^ was used to irradiate Au@CDs aqueous solutions with different mass concentrations at a distance of 2 cm from the dish. A highly sensitive electronic thermometer is used to monitor the temperature change of the system in real time.

**Monitoring of photoinduced enhanced peroxidase-like property of Au@CDs via UV-vis and SERS spectroscopy**

In this experiment, 30 μL TMB DMSO solution (15 mM), 30 μL H_2_O_2_ solution (0.01 M) and 30 μL above Au@CDs solution (1 mgmL^-1^) were mixed in 2.91 mL sodium acetate buffer (pH=4). The above reaction system was placed in a 4 mL volume quartz reaction cell, and the whole reaction was shaded or the reaction system was irradiated by an 808 nm laser with a power of 2 Wcm^-2^. At a certain interval, the mixed solution system was detected by UV-vis spectrum and SERS spectrum. The SERS experiments were performed with a LabRAM ARAMIS Smart Raman Spectrometer and the excitation source was the 633 nm line of a He/Ne laser.

### EPR measurement determining photoinduced enhanced peroxidase and glutathione oxidase-like properties of Au@CDs

100 μL DMPO wasdissolved in 900 μL DMSO solution and store in refrigerator at 4 °C.Then, the above 100 μL DMPO solution, 100 μL Au@CDs solution (1 mgmL^-1^) and 100 μL H_2_O_2_ solution (3×10^-3^ M) or GSH solution (3×10^-3^ M) were mixed, and the whole process was shaded in darkness. After shaking for 10 min, the above mixed solution was detected by EPR measurement.  Similarly, the above reaction system was subjected to 808 nm laser irradiation within 10 min for the determination of the photoinduced enhanced enzyme-like catalytic properties of Au@CDs, and the mixed solution system was detected by EPR measurement.

### In vitro antitumor effect

The study protocol (No. SY20212006) was approved by the laboratory animal center of Jilin University and was performed following the guidelines and regulations established by the tab of animal experimental ethical inspection, JLU.

To investigate the in vitro cytotoxicity of Au@CDs, 4T1 cells were treated by different concentration of Au@CDs (0, 20, 40, 80, 120 and 160 μgmL^-1^) for 24 h, and then detected cell viability using a CCK-8 assay kit.

To investigate the in vitro antitumor activity of Au@CDs based on the photoinduced enhanced enzyme-like catalytic properties, 4T1 cells were treated by Au@CDs (20 μgmL^-1^) for 24 h, and then exposed to 808 nm light irradiation (2 W cm^–2^) for 5 min, cell viability was detected using a CCK-8 assay kit. If H_2_O_2_ needs to be introduced in the experimental processes, 5 μL H_2_O_2_ (10^-3^ M) should be added to the medium 1h before illumination or cell viability test.

### Statistical analysis

All the data are presented as the mean ±SD. Unless stated otherwise, the experiments were performed in triplicate. The significance of the difference was determined through one-way analysis of variance (*p < 0.05, **p< 0.01).

### Calculation of photothermal conversion efficiency

According to previously reported method^3,4^, the photothermal conversion efficiency (η) of Au@CDs NPs was calculated, which is shown as follows:Based on the total energy balance for this system:

$$\begin{aligned} \sum_{i} m_{i}C_{p,i}\frac{dT}{dt}=Q_{\mathrm{NPs}}+Q_{s}-Q_{\mathrm{loss}}\#\left( 1 \right) \end{aligned}$$

where *T* represents the solution temperature. m and *C*_p_ represent the mass and heat capacity of solvent (water), respectively.

*Q*_NPs_is the photothermal energy input by Au@CDs NPs:

$$\begin{aligned} Q_{\mathrm{NPs}}=I\left( 1-{10}^{-A_{808}} \right)\eta\#\left( 2 \right) \end{aligned}$$

where *I*represents the laser power, *A*_808_ represents the absorbance of Au@CDs NPs at 808 nm, and *η* represents the photothermal conversion efficiency.

*Q*_s_ is the heat associated with the light absorbance of the solvent (water). *Q*_loss_ is the thermal energy lost to the surroundings:

$$\begin{aligned} Q_{\mathrm{loss}}=hA\Delta T\#\left( 3 \right) \end{aligned}$$

where *h* represents the heat transfer coefficient, *A* represents the surface area of the container, and *ΔT* represents the temperature change, which is defined as *T*-*T*_surr_ (*T* and *T*_surr_ are the solution temperature at cooling stage and surrounding temperature, respectively).

At the maximum steady-state temperature, the heat input is equal to the heat output, that is:

$$\begin{aligned} Q_{\mathrm{NPs}}+Q_{s}=Q_{\mathrm{loss}}=hA\Delta T_{\max}\#\left( 4 \right) \end{aligned}$$

where *ΔT*_max_ is the temperature change at the maximum steady-state temperature. According to the Eq.(2) and Eq.(4), the η can be determined as follows：

$$\begin{aligned} \eta=\frac{hA\Delta T_{\max}-Q_{s}}{I\left( 1-{10}^{-A_{808}} \right)}\#\left( 5 \right) \end{aligned}$$

And *hA* can be derived as follows:

$$\begin{aligned} \frac{d\frac{\Delta T}{\Delta T_{\max}}}{dt}=\frac{hA}{\sum m_{i}C_{p,i}}\left[ \frac{Q_{\mathrm{NPs}}+Q_{s}}{hA\Delta T_{\max}}-\frac{\Delta T}{\Delta T_{\max}} \right]\#\left( 6 \right) \end{aligned}$$

During cooling stage, the $Q_{\mathrm{NPs}}+Q_{s}=0$, thus changing Eq.(6) to:

$$\begin{aligned} dt=-\frac{\sum m_{i}C_{p,i}}{hA}\frac{d\frac{\Delta T}{\Delta T_{\max}}}{\frac{\Delta T}{\Delta T_{\max}}}\#\left( 7 \right) \end{aligned}$$

Integrating Eq.(7) gives the expression:

$$t=-\frac{\sum m_{i}C_{p,i}}{hA}ln\frac{\Delta T}{\Delta T_{\max}}$$

$$\begin{aligned} \frac{\Delta T}{\Delta T_{\max}}=\theta\#\left( 8 \right) \end{aligned}$$

Where*hA*canbe determined through the slope of the fitting line (131.6 s°C^-1^) in Figure 3d(the slope value of cooling time (*t*) versus the negative natural logarithm of the driving force temperature (*-lnθ*) obtained from the cooling stage), *m*is the mass of solution (1.0 g) and C is heat capacity (4.2 Jg^-1^) of the deionized water. Thus, the value of *hA* is deduced to be 32 mW°C^-1^.*Q*_s_is measured independently to be 25.2 mW using a quartz cuvette cell containing pure water. *I* is incident light power (2 Wcm^-2^, the spot diameter is 1.5 cm), A_808_ is the absorbance (0.9534 at the current concentration) of the used Au@CDs NPs at 808 nm.

The calculated photothermal conversion efficiency of Au@CDs NPs is about 39.96%, which is much higher than that of Au NPs with the same particle size prepared by Frens method (about 17%).

### Supporting Figures


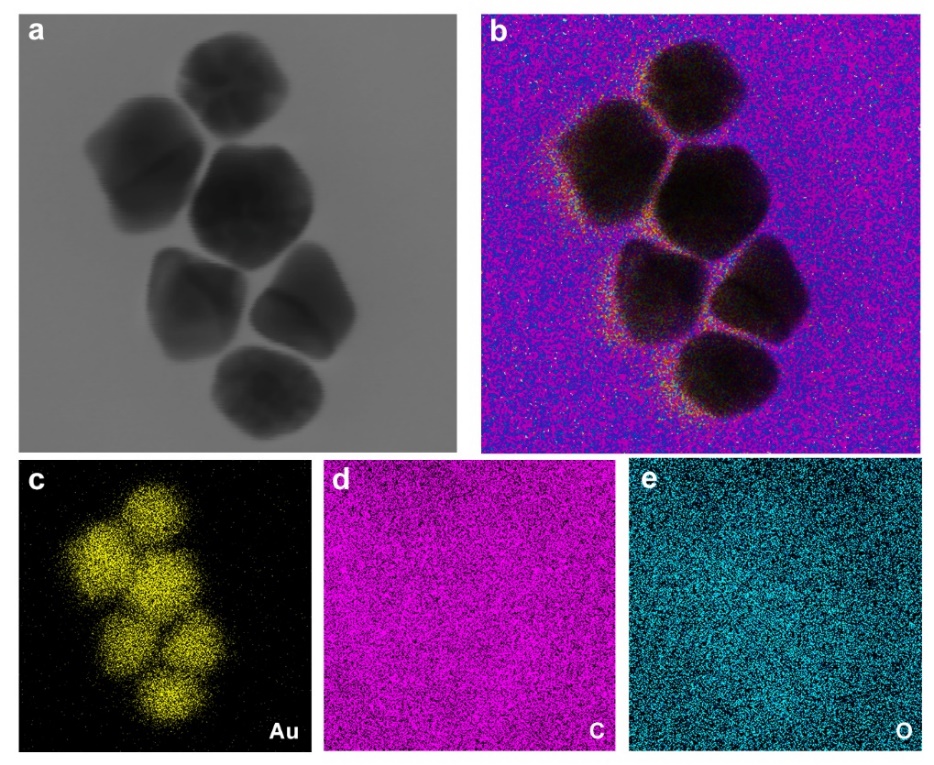


**Fig. S1**(a) TEM image and (b) the corresponding elemental mappings of (c) Au, (d) C and (e) O element of Au@CDs NPs on a copper grid with surface carbon film.


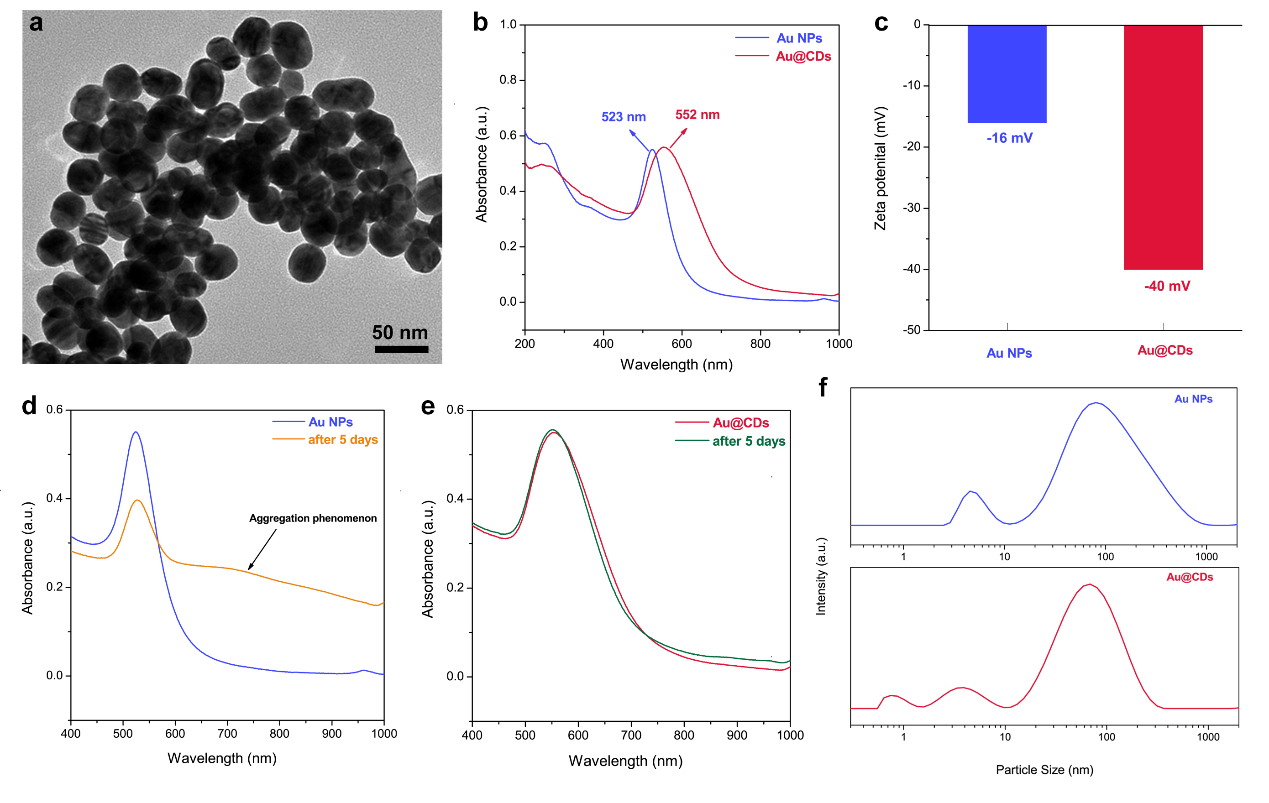


**Fig. S2** (a) TEM image of individual Au NPs with a mean diameter of ca. 40 nm. (b) UV-vis-NIR absorption spectra and (c) Zeta potential spectra of individual Au NPs and Au@CDs NPs in aqueous solution.(d,e) UV-vis absorption spectra of Au NPs and Au@CDs NPs in aqueous solution in the five days after preparation. (f) DLS tests for size distribution of individual Au NPs and Au@CDs NPs in aqueous solution.

**

**

**Fig. S3**FTIR spectrum of CDs.





**Fig.S4**Excitation-dependent PL behavior of CDs aqueous solution.

**
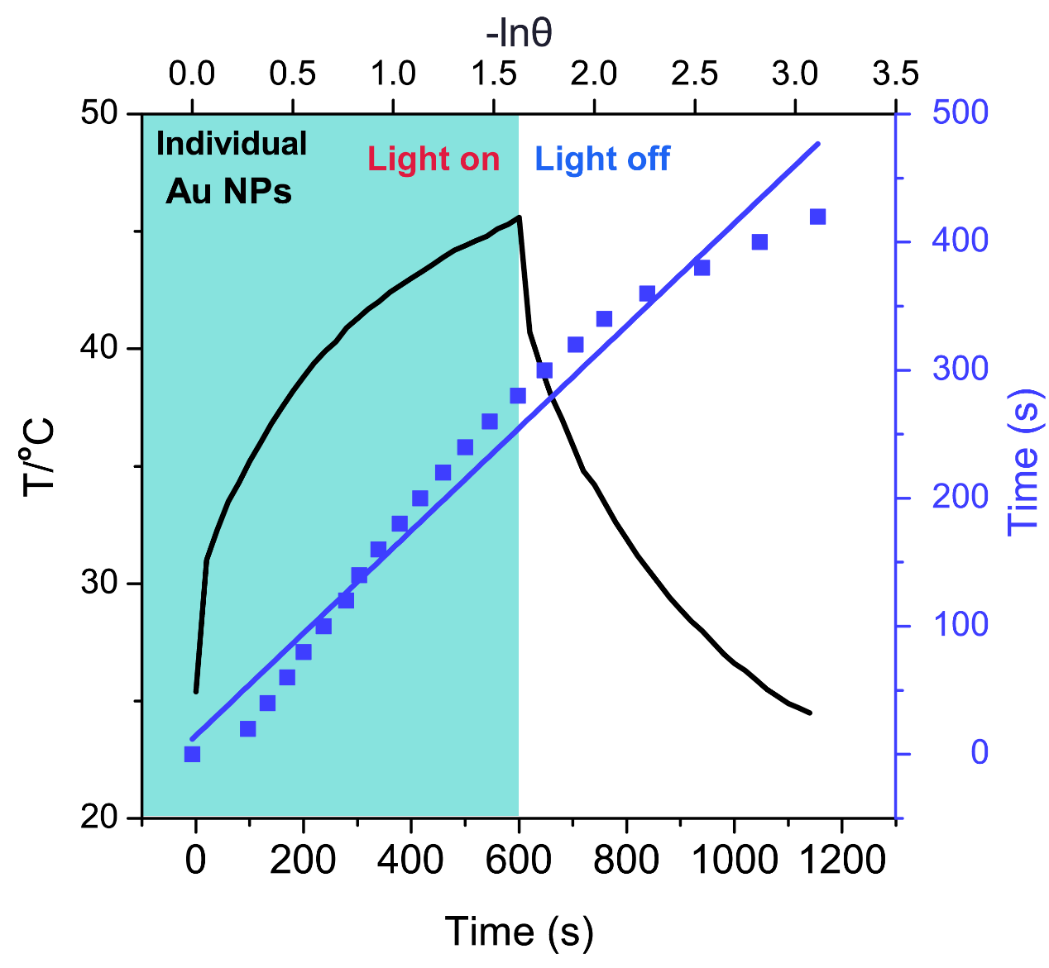
**

**Fig.S5**The heating and cooling curves of individual Au NPs for laser on/off and plot of cooling time versus the negative natural logarithm of the temperature driving force obtained from the cooling stage.


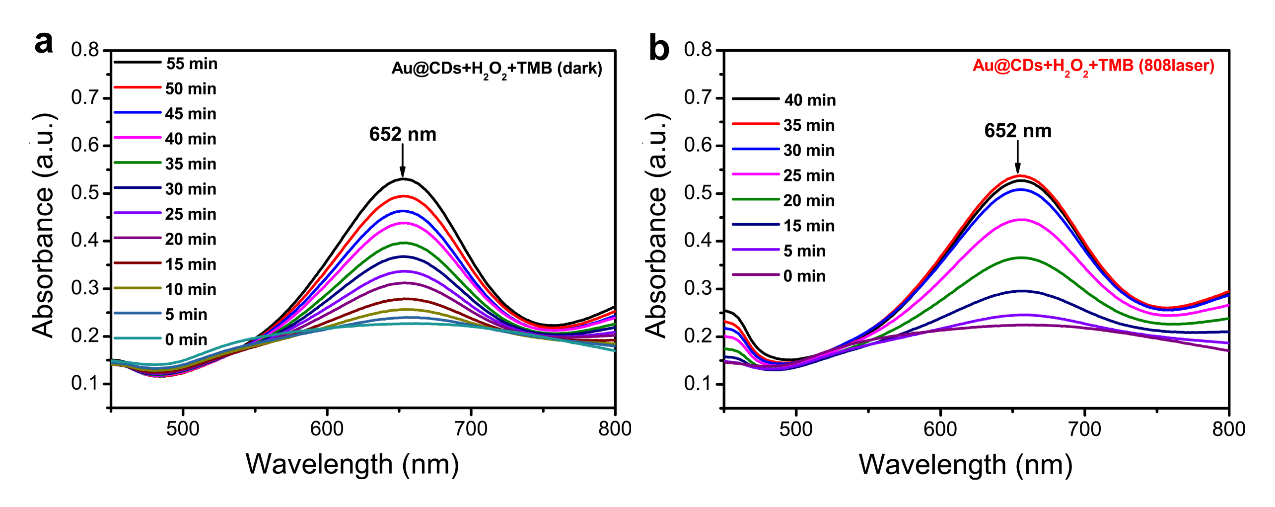


**Fig.S6**The absorbance versus wavelength plots at various times for the oxidation of TMB (a) in darkness and (b) under 808 nm laser irradiation.


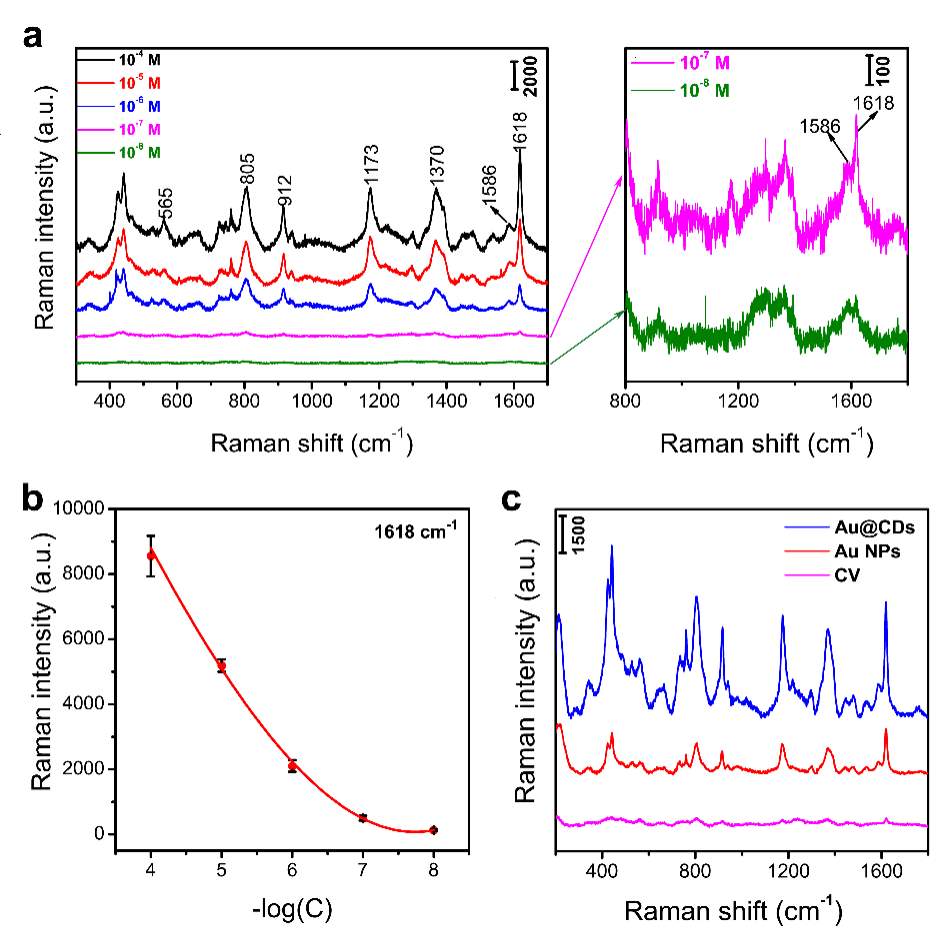


**Fig.S7**(a) SERS spectra of CV molecules with different concentrations using Au@CDs as SERS substrates in aqueous suspension, and (b) their corresponding calibration curve of peak intensity versus CV concentration at 1618 cm^-1^. (c) SERS spectra of CV using individual Au NPs and the Au@CDs as SERS substrates in aqueous suspension.


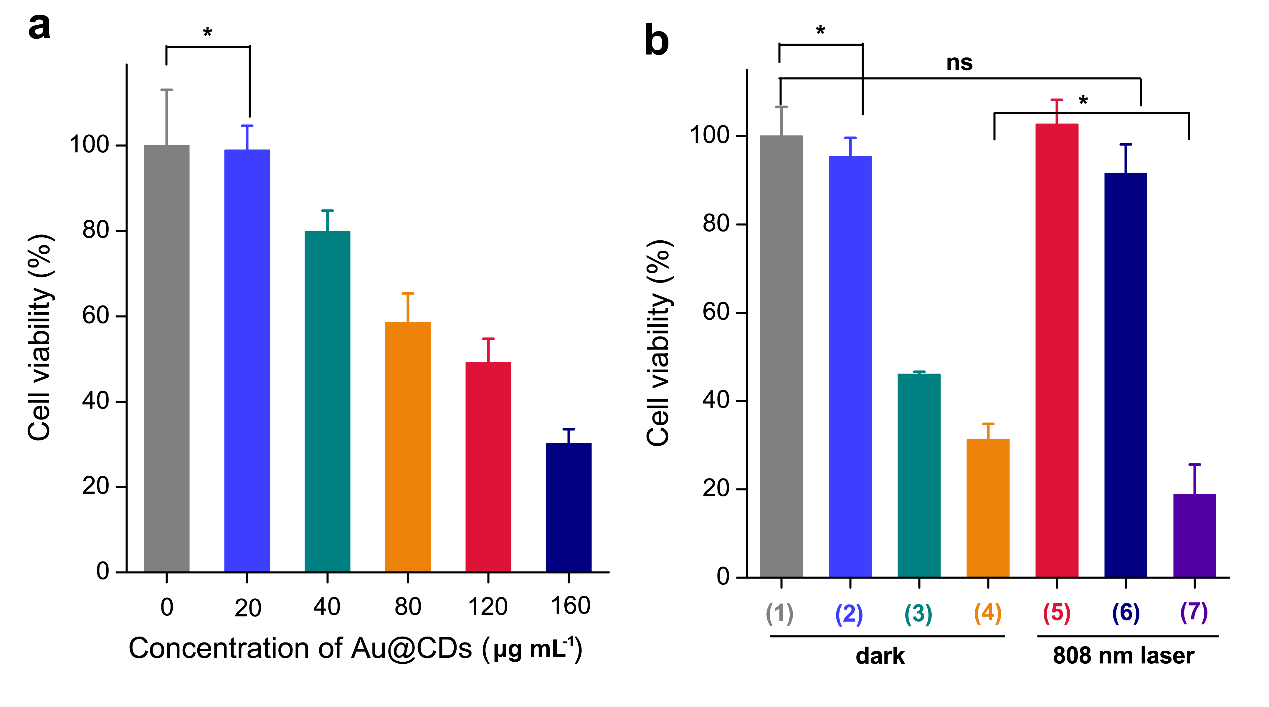


**Fig.S8**(a) Cell viability of tumor cells (4T1) in the presence of Au@CDs with different concentrations in dark. (b) Cell viability of tumor cells (4T1) after different treatments: (1) Control, (2) Au@CDs, (3) H_2_O_2_, (4) Au@CDs + H_2_O_2_, (5) NIR, (6) Au@CDs + NIR, (7) Au@CDs + H_2_O_2_ +NIR.


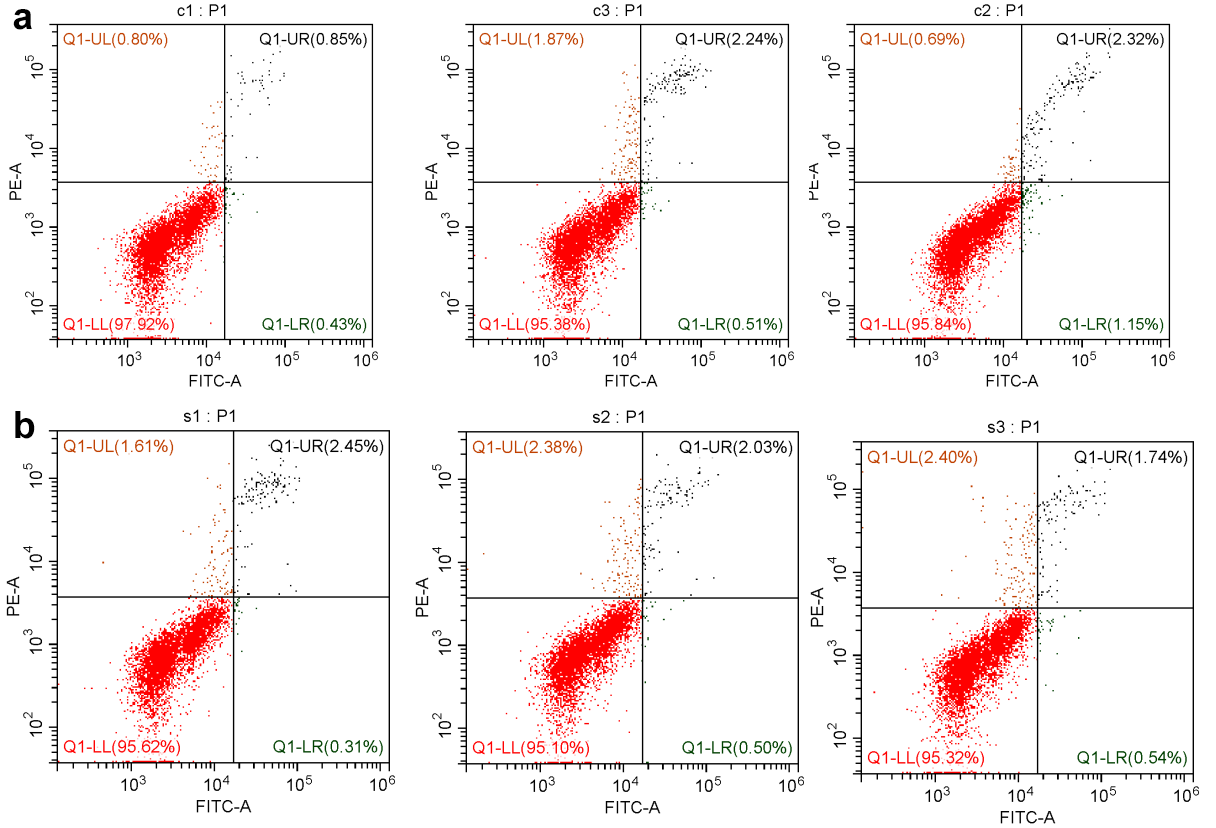


**Fig.S9**(a)Apoptosis ratios of 4T1 cells treated with (a) PBS and (b) Au@CDs + H_2_O_2_ + NIR determined by flow cytometry using the Annexin V-FITC/PI staining kit. Repeat three times for each group.


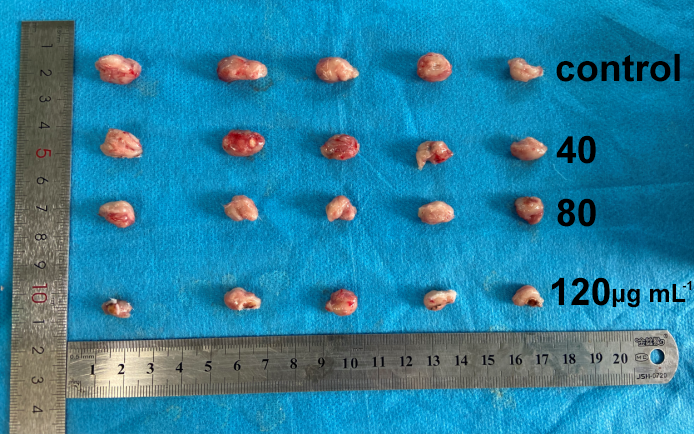


**Fig.S10**Photographs of the tumor after PDT/PTT co-therapy with Au@CDs of different concentrations.


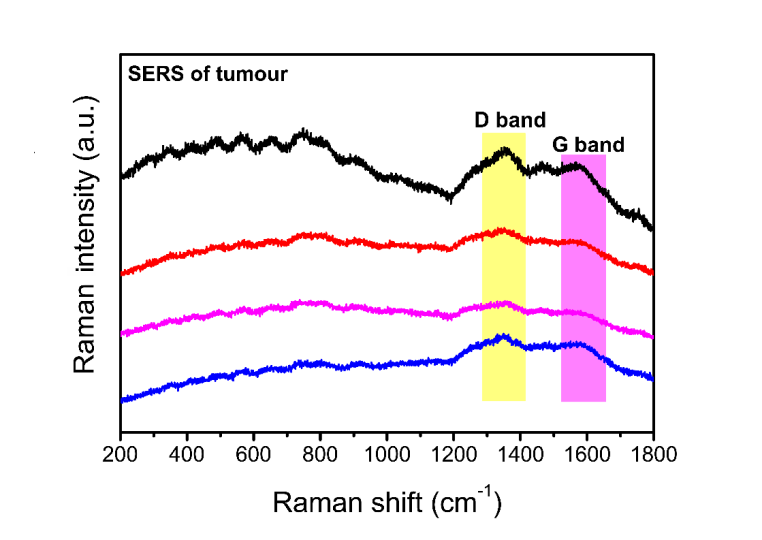


**Fig.S11**SERS spectra of Au@CDs in tumor.

### Supporting Tables

**Table S1** Detailed information of different atom types of CDs.

| **Atom** | **Atom types** | **Binding energy (eV)** | **Peak area** | **Content** |
| --- | --- | --- | --- | --- |
| **C1s** | C-C/C=C | 284.6 | 39200 | 59.6% |
|  | C-O | 286.1 | 11822 | 18% |
|  | C=O | 287.7 | 9450 | 14.4% |
|  | O=C-O | 288.8 | 5222 | 8% |
| **O1s** | C=O | 531.5 | 22536 | 54.8% |
|  | C-O | 533.2 | 18546 | 44.2% |

**Table S2** Detailed information of different atom types of Au@CDs.

| **Atom** | **Atom types** | **Binding energy (eV)** | **Peak area** | **Content** |
| --- | --- | --- | --- | --- |
| **C1s** | C-C/C=C | 284.6 | 36092 | 85.7% |
|  | C-O | - | - | - * |
|  | C=O | 286.7 | 3963 | 9.4% |
|  | O=C-O | 288.3 | 2064 | 4.9% |
| **O1s** | C=O | 531.6 | 43771 | 93.6% |
|  | C-O | 533.5 | 3001 | 6.4% |

* The amount of C-O has decreased so dramatically that we didn't list it in the table of C1s.

**The probable formation mechanism of Au@CDs：**

Briefly, CDs with abundant reductive groups exhibited the potential ability of directly reducing Au^3+^ to Au^0^. Based on the results of XPS spectrum (Figure 2 in the manuscript), the types of functional groups on the CDs surface are mainly oxygen-containing functional groups, containing of -COOH, C=O, and -OH. In the synthesis process of Au@CDs, CDs acts as an electron donor to provide electrons and the electron outflow positions are the particular functional groups addresses on the surface. According to some reports, the electron-donor capacity of oxygen-containing functional groups on the surface of carbon quantum dots varies greatly^5^. As a matter of fact, -OH has the strongest the electron-donor ability than C=O and -COOH^5,6^. Therefore, we believe that the CDs' ability to be a reducing agent mostly comes from the hydroxyl groups on the surface, which are removed during the reaction, resulting in a significant decrease of C-O (from C-OH) in content after the formation of Au@CDs.

In addition, as a capping agent^7^, the chemical environment of C=O of CDs will undergo a little change after linking with Au. That's probably why there's a chemical shift of C=O from 287.6 eV to 286.8 eV). Furthermore, as mentioned above, the content of C=O and -COOH have a bit decline after the reaction process compared with that of C-OH, which can be resulting from the interactions between the them with Au cores (Figure 2d). To clearly illustrate the contents of C-O, C=O and -COOH, we have provided the peak areas of these functional groups and calculated their contents, which have been illustrated in Table S1 and S2.

**Table S3** Comparative analysis of photothermal activities under 808 nm laser irradiation based on different types of plasmon-based nanomaterials.

| **Material** | **Laser power (Wcm^-2^)** | **Photothermal conversion efficiency (%)** | **Reference** |
| --- | --- | --- | --- |
| **Au@CDs**  **Au_2_Pt-PEG-Ce_6_**  **Fe_3_O_4_/Au NCs@LCPAA-TPP**  **MoO_3_-Ag-PEG-MnO_2_** | 2.0 | 39.96 | this work |
|  | 1.0 | 31.5 | ^8^ |
|  | 1.0 | 25.4 | ^9^ |
|  | 1.2 | 37.0 | ^10^ |
| **CuFe NS**  **MHPCNs-SS-PGA-FA** | 2.0 | 27.82 | ^11^ |
|  | 1.5 | 36.0 | ^12^ |
| **Au NCs@PMLE/Ca^2+^** | 1.0 | 24.3 | ^13^ |
| **TTPY-Py@Au NR** | 1.0 | 30.0 | ^14^ |
| **Au_4_Cu_4_/Au_25_ NCs@LiP** | 1.0 | 31.73 | ^15^ |
| **MSNR@Au-TPPS_4_** | 1.5 | 32.01 | ^16^ |
| **Au-Bi-GSH@IR808** | 0.5 | 34.2 | ^17^ |

**Table S4** Detailed information of Raman shifts of crystal violet^18^.

| **Raman shift (cm^-1^)** | **Irreducible representation** | **Assignment** |
| --- | --- | --- |
| **420** | E | δ (CNC) / δ (CC_center_C) |
| **441** | A_1_ | δ (CNC) |
| **525** | E | δ (CNC) |
| **565** | E | γ (CCC) / δ (CNC) / δ (CC_center_C) |
| **606** | A_1_ | δ (CCC) / δ (CNC) / ν_s_ (CC_center_C) |
| **725** | E | ν (CN) |
| **763** | A_1_ | ν_s_ (CC_center_C) / ν (CN) |
| **805** | E | δ (CH)_ring_ |
| **912** | E | δ (CC_center_C) |
| **1173** | E | ν_as_ (CC_center_C) |
| **1299** | E | ν_as_ (CC_center_C) / δ (CCC)_ring_ / δ (CH) |
| **1370** | E | ν_as_ (CC_center_C) / δ (CCC)_ring_ / δ (CH) |
| **1445** | E | δ_as_ (CH_3_) |
| **1476** | E | δ_as_ (CH_3_) |
| **1535** | E | ν (C_ring_N) /δ_s_ (CH_3_) |
| **1586** | E | ν (CC)_ring_ |
| **1618** | A_1_ | ν (CC)_ring_ |

ν, stretching (s, symmetric; as, asymmetric); δ, bending; γ, out-of-plane deformation (respect to the benzene ring).

### Reference

1 Zhu, S. J. *et al.* Highly photoluminescent carbon dots for multicolor patterning, sensors, and bioimaging. *Angewandte Chemie International Edition***52**, 3953-3957, (2013).

2 Xia, H. B.*et al.* Revitalizing the frens method to synthesize uniform, quasi-spherical gold nanoparticles with deliberately regulated sizes from 2 to 330 nm. *Langmuir***32**, 5870-5880, (2016).

3 Zeng, K. *et al.* Coordination nanosheets of phthalocyanine as multifunctional platform for imaging-guided synergistic therapy of cancer. *ACS Applied Materials & Interfaces***11**, 6840-6849, (2019).

4 Ji, M. *et al.* Structurally well-defined Au@Cu_2-x_S core–shell nanocrystals for improved cancer treatment based on enhanced photothermal efficiency. *Advanced Materials***28**, 3094-3101, (2016).

5 Zhang, T. Y. *et al.* Regulation of functional groups on graphene quantum dots directs selective CO_2_ to CH_4_ conversion. *Nature Communication***12**, 5265, (2021).

6 Zhang, P. *et al.* Insight into metal-free carbon catalysis in enhanced permanganate oxidation: changeover from electron donor to electron mediator. *Water Research***219**, 118626, (2022).

7 Chen, M. M. *et al.* Tuning the aggregation of silver nanoparticles with carbon dots for the surface-enhanced Raman scattering application. *Carbon***185**, 442-448, (2021).

8 Wang, M. *et al.* Au_2_Pt-PEG-Ce_6_ nanoformulation with dual nanozyme activities for synergistic chemodynamic therapy / phototherapy. *Biomaterials***252**, 120093, (2020).

9 Yang, Y. *et al.* Design of the tumor microenvironment-multiresponsive nanoplatform for dual-targeting and photothermal imaging guided photothermal/photodynamic/chemodynamic cancer therapies with hypoxia improvement and GSH depletion. *Chemical Engineering Journal***441**, 136042, (2022).

10 Wu, F. *et al.* MoO_3-x_ nanosheets-based platform for single NIR laser induced efficient PDT/PTT of cancer. *Journal of Controlled Release***338**, 46-55, (2021).

11 Liu, Y. *et al.* All-in-one theranostic nanoagent with enhanced reactive oxygen species generation and modulating tumor microenvironment ability for effective tumor eradication. *ACS Nano***12**, 4886-4893, (2018).

12 Wu, F. *et al.* Triple stimuli-responsive magnetic hollow porous carbon-based nanodrug delivery system for magnetic resonance imaging-guided synergistic photothermal/chemotherapy of cancer. *ACS Applied Materials & Interfaces***10**, 21939-21949, (2018).

13 Yang, Y. M. *et al.* A multi-responsive Au NCs@PMLE/Ca^2+^ antitumor hydrogel formed in situ on the interior/surface of tumors for PT imaging-guided synergistic PTT/O^2-^ enhanced PDT effects. *Nanoscale***14**, 7372-7386, (2022).

14 Song, N. *et al.* Pillar[5]arene-modified gold nanorods as nanocarriers for multi-modal imaging-guided synergistic photodynamic-photothermal therapy. *Advanced Functional Materials***31**, 2009924, (2021).

15 Liu, X. *et al.* Self-assembled Au_4_Cu_4_/Au_25_ NCs@liposome tumor nanotheranostics with PT/fluorescence imaging-guided synergetic PTT/PDT. *Journal of Materials Chemistry B***9**, 6396-6405, (2021).

16 Yang, S. *et al.* Rodlike MSN@Au nanohybrid-modified supermolecular photosensitizer for NIRF/MSOT/CT/MR quadmodal imaging-guided photothermal/photodynamic cancer therapy. *ACS Applied Materials & Interfaces***11**, 6777-6788, (2019).

17 Jia, P. P. *et al.* Integration of IR-808 and thiol-capped Au–Bi bimetallic nanoparticles for NIR light mediated photothermal/photodynamic therapy and imaging. *Journal of Materials Chemistry B***9**, 101-111, (2021).

18 Kleinman, S. L. *et al.* Single-molecule surface-enhanced Raman spectroscopy of crystal violet isotopologues: theory and experiment. *Journal of the American Chemical Society***133**, 4115-4122, (2011).
